# Extrapyramidal adverse events and anticholinergics use after the long-term treatment of patients with schizophrenia with the new long-acting antipsychotic Risperidone ISM^®^: results from matching-adjusted indirect comparisons *versus* once-monthly formulations of Paliperidone palmitate and Aripiprazole monohydrate in 52-week studies

**DOI:** 10.1186/s12991-023-00464-z

**Published:** 2023-09-02

**Authors:** Pedro Sánchez, Cecilio Álamo, Marcos Almendros, Max Schlueter, Anastasios Tasoulas, Javier Martínez

**Affiliations:** 1 Hospital of Zamudio. Bizkaia Mental Health Network. Osakidetza Basque Health Service, Bilbao, Spain; 2 Faculty of Health Sciences. Department of Medicine, Deusto University, Bilbao, Spain; 3 Department of Biomedical Sciences, Alcalá University. Alcalá de Henares, Madrid, Spain; 4 Medical Department, Laboratorios Farmacéuticos ROVI, S.A, Calle Alfonso Gómez, 45B. 28037 Madrid, Spain; 5 IQVIA, London, UK; 6 IQVIA, Athens, Greece

**Keywords:** Schizophrenia, Risperidone, Matching-adjusted indirect comparison, MAIC, Extrapyramidal symptom, Anticholinergics

## Abstract

**Background:**

Risperidone ISM^®^ is a newly developed long-acting injectable (LAI) treatment for schizophrenia in adults. In the absence of head-to-head comparisons with other similar antipsychotics, the objective of this study was to generate indirect evidence of some aspects of the safety and tolerability of Risperidone ISM compared to other LAI antipsychotics for treatment of patients with schizophrenia in the maintenance treatment setting.

**Methods:**

A literature review was conducted systematically to identify maintenance treatment studies reporting safety and tolerability outcomes for LAI antipsychotic therapies. Following an assessment of between-trial heterogeneity, a matching-adjusted indirect comparison (MAIC) was performed to account for between-trial imbalances in patient characteristics and to generate comparative evidence for safety and tolerability endpoints.

**Results:**

The analysis showed that incidence of extrapyramidal symptoms (EPS) was found to be numerically, but not statistically significantly, lower in patients receiving Risperidone ISM than in those receiving Paliperidone palmitate (PP) (OR [95% CI] 0.63 [0.29, 1.38], *p* = 0.253) and statistically significantly lower than with Aripiprazole monohydrate once-monthly (AOM) (OR [95% CI] 0.25 [0.12, 0.53], *p* < 0.001). Use of anticholinergic agents for the alleviation of EPS was also shown to be significantly lower in Risperidone ISM patients than in those receiving PP (OR [95% CI] 0.29 [0.10, 0.83], *p* = 0.021) or AOM (OR [95% CI] 0.01 [0.003, 0.06], *p* < 0.001), suggesting a superior tolerability profile for clinically relevant EPS. Results from the sensitivity analyses comparing stabilized and stable patients receiving Risperidone ISM to those receiving AOM yielded similarly favorable conclusions in line with the base case analyses.

**Conclusions:**

This MAIC is consistent with the safety and tolerability results obtained during the PRISMA-3 clinical trial in the long-term treatment of schizophrenia and suggests a favorable safety and tolerability profile in terms of EPS incidence and anticholinergic agent use, relative to other antipsychotic therapies used for treatment of patients with schizophrenia in the maintenance setting.

**Supplementary Information:**

The online version contains supplementary material available at 10.1186/s12991-023-00464-z.

## Background

Schizophrenia is a severe, chronic mental disorder [1] and among the leading global causes of disability [2]. It is associated with a substantial reduction in patient quality of life and a substantially reduced lifespan [3]. Despite their efficacy in managing schizophrenia, current antipsychotics are associated with several motor adverse effects broadly classified as extrapyramidal symptoms (EPS), which can reduce overall treatment benefit [4], induce medical costs [5], and are considered a cause of non-adherence due to their negative impact on patient quality of life [6]. Second-generation (atypical) antipsychotics have shown promise compared to first-generation treatments, as they cause fewer EPS and have generally demonstrated more favorable outcomes in terms of safety and tolerability [7]. However, the risk of EPS persists even for second-generation treatments, particularly when administered at higher doses [6]. Anticholinergic agents are the primary treatment for antipsychotics-induced EPS, but their prophylactic and long-term use is discouraged by clinicians as they are known to cause a variety of side-effects, such as blurred vision, tachycardia, hallucinations, tardive dyskinesia and generalized cognitive impairment, as well as, possibly, a higher risk of dementia [8–12].

In addition to oral antipsychotics, long-acting injectable (LAI) formulations of antipsychotics have been developed that relieve patients from the need to receive their medication daily [13–15] and have thus improved treatment adherence and disease management [16] as well as superior outcomes in comparison to their orally administered counterparts [17, 18]. However, unmet clinical need persists with regards to LAIs achieving adequate therapeutic plasma levels at treatment initiation [14, 19, 20], as well as gaps in the evidence of and opportunities for improvements in their long-term safety profiles [17, 21].

As an alternative safe and efficacious treatment option, the European Union recently authorized Risperidone ISM^®^, a new LAI formulation of risperidone, which is administered once-monthly (every 4 weeks) and provides immediate and sustained therapeutic drug plasma levels without the need for oral risperidone supplementation or loading doses [19, 22]. The efficacy and safety of Risperidone ISM was evaluated in the PRISMA-3 clinical trial, which included a 12-week placebo-controlled double-blind (DB) phase [23] (NCT03160521) that randomized patients to placebo or Risperidone ISM 75 or 100 mg. The DB phase was followed by a 1-year open-label extension (OLE) phase [24] (NCT03870880) in which patients on prior treatment with placebo were randomized to Risperidone ISM (“unstable patients”), while patients on prior treatment with Risperidone ISM continued to receive the same regimen (“stabilized patients”). In addition, the OLE enrolled a third cohort of stable de novo patients who had not participated in the DB phase and met eligibility criteria (“stable patients”). The OLE demonstrated Risperidone ISM to be an effective, safe, and well-tolerated long-term treatment of schizophrenia in adults, regardless of patient baseline disease severity, prior Risperidone ISM treatment during an acute exacerbation, or treatment switching from stable doses of oral risperidone [24]. However, no direct or indirect comparisons of Risperidone ISM to other LAI antipsychotics have been performed up to now.

The aim of this study is to compare Risperidone ISM as a maintenance therapy to other common [25, 26] once-monthly LAI formulations of atypical antipsychotics such as Aripiprazole monohydrate once-monthly (AOM) and Paliperidone palmitate (PP) for the safety and tolerability endpoints of EPS incidence and use of anticholinergic agents, by means of a matching-adjusted indirect comparison (MAIC).

## Methods

### Literature review

A literature review was conducted systematically in September 2020 using the PICOS (population, intervention, comparator, outcome, study design) eligibility criteria to identify English-language publications reporting relevant safety and tolerability outcomes for comparator antipsychotics PP and AOM in patients with schizophrenia treated in the maintenance setting. Searches were run in the EMBASE, MEDLINE, Cochrane Central Register of Controlled Trials (CCTR) and Journals@Ovid databases via the Ovid platform. The terms used for the search strategy are provided in the Additional file 1. Retrieved citations were screened first at title and abstract level and second at full text level. Articles were cross-checked with a 2020 Cochrane systematic literature review (SLR) in schizophrenia [27] to ensure no relevant studies were missed. One researcher conducted the screening and data extraction, whereas a second reviewer cross-checked the extracted data against the original sources.

At first-stage screening, five publications of interest were identified: Hough 2010 [28] (NCT00111189, DB phase results), Gopal 2010 [29] (NCT00111189, OLE phase results from the same trial), Kane 2012 [30] (NCT00705783), Fleischhacker 2012 [31] (NCT00210717), Fleischhacker 2014 [32] (NCT00706654), and Naber 2015 [33] (NCT01795547). Key characteristics of the identified trials are presented in detail in Additional file 1: Table S1.

### Feasibility assessment

The identified trials were examined with regards to design and population heterogeneity. While overall key inclusion/exclusion criteria were similar (Additional file 1: Table S2), the assessment concluded that only two of the five studies were eligible for evidence synthesis due to considerations related to study design and disease characteristics. As duration of sustained schizophrenia treatment has an important effect on patient stabilization [34, 35], this is likely to introduce bias when comparing studies of differing durations. Hence, the Hough 2010 DB (24 weeks), Fleischhacker 2014 (38 weeks) and Naber 2015 (28 weeks) studies were discarded due to their substantially shorter durations compared to PRISMA-3 OLE (52 weeks). The Fleischhacker 2012 study was also discarded as it exclusively recruited acutely symptomatic (i.e., unstable) patients, whereas PRISMA-3 OLE included 25.6% patients with unstable disease. Selected comparator studies were hence narrowed down to Gopal 2010 [29], and Kane 2012 [30]. Table 1 provides an overview of their patients’ characteristics. Overall, the three studies were considered adequately similar for inclusion in the subsequent indirect comparisons.

**Table 1:** Patient baseline characteristics in included studies

Study (reference)	PRISMA-3 OLE [24]				Gopal 2010 [29]	Kane 2012 [30]
Patient cohort	Unstable^a^ *N* = 55	Stabilized^b^ *N* = 119	Stable^c^ *N* = 41	All *N* = 215	All *N* = 388	AOM arm *N* = 269
Age [Mean (SD)]	38.2 (10.0)	40.3 (11.5)	36.7 (9.9)	39.1 (10.9)	37.3 (10.8)	40.1 (11.0)
Sex [n (%)]						
Male	31 (56.4)	76 (63.9)	24 (58.5)	131 (60.9)	209 (54.0)	162 (60.2)
Female	24 (43.6)	43 (36.1)	17 (41.5)	84 (39.1)	179 (46.0)	107 (39.8)
Race [n (%)]						
White	44 (80.0)	97 (81.5)	41 (100.0)	182 (84.7)	269 (69.0)	152 (56.5)
Black	11 (20.0)	21 (17.6)	0 (0.0)	32 (14.9)	41 (11.0)	59 (21.9)
Asian	0 (0.0)	1 (0.8)	0 (0.0)	1 (0.5)	72 (19.0)	45 (16.7)
Other	0 (0.0)	0 (0.0)	0 (0.0)	0 (0.0)	6 (2.0)	13 (4.8)
Age at diagnosis [Mean (SD)]	27.7 (7.5)	28.5 (8.5)	27.9 (7.7)	28.2 (8.1)	26.8 (8.5)	25.8 (8.3)
PANSS total score [Mean (SD)]	82.3 (12.2)	70.0 (14.3)	60.3 (8.2)	71.3 (14.8)	58.2 (18.2)	54.5 (NR)
CGI-S score [Mean (SD)]	4.1 (0.9)	3.4 (0.9)	2.8 (0.5)	3.5 (0.9)	3.0 (median)	2.9 (NR)
BMI [Mean (SD)]	27.6 (4.7)	27.0 (4.9)	25.5 (3.7)	26.9 (4.7)	27.2 (5.6)	28.1 (6.9)

*AOM* aripiprazole monohydrate once-monthly, *BMI* body mass index, *CGI-S* clinical global impressions—severity scale, *DB* double-blind, *NR* not reported, *OLE* open-label extension, *PCB* placebo, *PANSS* positive and negative syndrome scale, *SD* standard deviation

^a^ Unstable patients, PCB rollover patients from the DB phase of PRISMA-3 study who were randomly assigned to Risperidone ISM at dose of either 75 or 100 mg in the OLE phase

^b^ Stabilized patients, patients treated with Risperidone ISM in the DB phase who continued to receive monthly Risperidone ISM in the OLE phase at the same dose (75 or 100 mg) as during the DB phase

^c^ Stable patients, newly enrolled patients (de novo) who were on a previous stable maintenance dose of oral risperidone

### Outcomes

Given its clinical significance, incidence of EPS was selected as the safety outcome of this analysis. As per the *Medical Dictionary for Regulatory Affairs* (MedDRA) Standardised Medical Queries (SMQ) “broad” definition, EPS is a basket term comprising akathisia, tremor, restlessness, and extrapyramidal disorder component outcomes. While no clear definitions of EPS are provided in the comparator studies, it is known to have included tremors in Gopal 2010, and akathisia events in Kane 2012.

Use of anticholinergic agents was selected as the tolerability outcome of this analysis as a low rate of use would imply a favorable tolerability profile for the associated antipsychotic drug, lower exposure to the risk of negative anticholinergic side-effects, and a lower overall drug burden. While the Kane 2012 study explicitly mentions the term “anticholinergic agents”, the Gopal 2010 study mentions “anti-EPS medication”, which was assumed to imply anticholinergic agents.

### Populations

The population of Gopal 2010 consisted of three patient cohorts treated with PP: patients who rolled over from PCB (PCB/PP group), patients who remained on PP (PP/PP group), and patients who were in the transition/maintenance phase before entering the OLE (TM/PP group). Based on their positive and negative syndrome scale (PANSS) [36] total scores at OLE entry [29], the PCB/PP group had the most severe symptoms, followed by the PP/PP group and the TM/PP group, the latter of which had the least heavy symptoms. These cohorts were considered similar to the unstable, stabilized, and stable cohorts of PRISMA-3 OLE respectively, so all safety and tolerability comparisons were performed using the full populations from those two studies.

For comparisons with AOM (as informed by Kane 2012), the full population of the PRISMA-3 OLE was used in the base case, but scenario analyses (fully presented in the Additional file 1) were additionally performed using only the stable and stabilized cohorts of the OLE. This approach was adopted based on the rationale that the 12-week stabilization requirement in Kane 2012 prior to randomization may have resulted in a patient population with less severe symptoms than expected in clinical practice [30], as a result of which comparisons with the non-unstable cohorts of PRISMA-3 OLE would be more appropriate.

### Statistical analysis

MAICs reduce bias that is caused by between-trial imbalances in patient characteristics that influence treatment effects or clinical outcomes (termed “effect modifiers” and “prognostic factors”, respectively), using individual patient data (IPD) from the pivotal trial, and reported aggregate-level data from the comparator trials. This is achieved by re-weighting the IPD to match patients’ baseline characteristics to those of the comparator populations at the aggregate level, and then proceeding to incorporate those weights into the estimation of relative treatment effects. This process produces relative effect estimates that we would expect to observe if the comparator trials included an arm of the pivotal trial’s experimental intervention. The methodology is well-established and in line with the guidelines of the UK’s National Institute for Health and Care Excellence (NICE) [37], as well as with EUnetHTA methodological guidance [38].

As no common treatments existed between the compared trials, unanchored MAICs were conducted, whereby between-trial relative effects were derived by comparing the absolute effects of the trial arms of interest. Relative effects were estimated in the form of odds ratios (OR), and variances were derived using robust sandwich estimators. Precision of adjusted estimates was evaluated using the effective sample size (ESS), which was calculated in line with NICE DSU guidance [37].

Baseline characteristics eligible for matching were those mutually reported across all compared studies: age, sex, race group, body mass index (BMI), age at schizophrenia diagnosis, baseline PANSS and clinical global impressions—severity scale (CGI-S) [39] total scores. Selection of variables for matching was based on statistical testing: logistic regression models were fitted using each variable as a predictor on the outcome of interest, and likelihood ratio tests (LRTs) compared these models to null, intercept-only models. Variables where the LRT returned a statistically significant *p*-value were considered prognostic factors and selected for adjustment in the MAIC. Taking a conservative approach that accounts for low power, a 10% significance threshold was preferred over the conventional 5%. Tests were performed on the full PRISMA-3 OLE population (*N* = 215) and the pooled stabilized and stable cohorts (*N* = 160). As all patients in PRISMA-3 OLE received Risperidone ISM, no treatment variable could be defined, and thus no effect modification could be tested for.

Both adjusted and unadjusted relative effects were estimated for each comparison. No missing data were observed in the IPD and thus no imputation was necessary. Statistical analysis was conducted using the R statistical software [40] and a variety of supportive packages [41–47], through the RStudio interface [48].

## Results

### Population matching

Statistical testing (detailed results in Additional file 1: Table S3) determined that baseline PANSS total score, CGI-S score, race group and age were prognostic factors for the tolerability outcome of anticholinergic agent use at the 10% significance level. Age tested as a prognostic factor only in the pooled PRISMA-3 OLE stabilized and stable cohorts, but as a conservative approach it was matched in the base case analysis that compared the full PRISMA-3 OLE population as well. No variable tested as a prognostic factor for the safety outcome of EPS, thus comparisons proceeded without population adjustment for this outcome.

Race group was matched as a binary variable where patients other than white, namely “non-white” patients, were pooled together in a single category. This was necessary because the PRISMA-3 OLE included only one Asian patient, and no category of “other”. In contrast, the Gopal 2010 and Kane 2012 studies had substantially more Asian patients, as well as a racial category of “other”. Thus, pooling was pursued to circumvent obstacles related to lack of population characteristics overlap, and to avoid imbalances that would severely reduce the ESS in the subsequent adjusted comparisons.

Tables 2 and 3 present baseline characteristics pre- and post-matching, against the comparator studies of Gopal 2010 [29] and Kane 2012 [30], respectively.

**Table 2:** Characteristics matching in Risperidone ISM versus Paliperidone palmitate comparison, for the tolerability base case analysis

Study	PRISMA-3 OLE (Full population)		Gopal 2010 [28]
Intervention	Risperidone ISM		PP
Characteristic	Pre-matching (*N* = 215)	Post-matching (ESS = 42)	(*N* = 388)
Age [Mean (SD)]	39.1 (10.9)	37.3 (10.8)	37.3 (10.8)
Baseline PANSS total score [Mean (SD)]	71.3 (14.8)	58.2 (18.2)	58.2 (18.2)
Baseline CGI-S score [Median]	3.0	3.0	3.0
Race: white [%]	84.7	69.0	69.0

*CGI-S* clinical global impressions—severity scale, *ESS* effective sample size, *MAIC* matching-adjusted indirect comparison, *OLE* open-label extension, *PANSS* positive and negative syndrome scale, *PP* paliperidone palmitate, *SD* standard deviation

**Table 3:** Characteristics matching in Risperidone ISM versus Aripiprazole monohydrate once-monthly comparison, for the tolerability base case analysis

Study	PRISMA-3 OLE (Full population)		Kane 2012 [30] (AOM arm)
Intervention	Risperidone ISM		AOM
Characteristic	Pre-matching (*N* = 215)	Post-matching (ESS = 26)	(*N* = 269)
Age [Mean (SD)]	39.1 (10.9)	40.1 (11.0)	40.1 (11.0)
Baseline PANSS total score [Mean]	71.3	54.5	54.5
Baseline CGI-S total score [Mean]	3.5	2.9	2.9
Race: white [%]	84.7	56.5	56.5

*AOM* aripiprazole monohydrate once-monthly, *CGI-S* clinical global impressions—severity scale, *ESS* effective sample size, *OLE* open-label extension, *PANSS* positive and negative syndrome scale, *SD* standard deviation

Characteristics of PRISMA-3 OLE patients were exactly matched to the aggregate statistics reported in the comparator trials. The reductions from an initial sample size of 215 to an ESS of 42 for the PP and 26 for the AOM comparison are substantial, reflecting the impact of the characteristics’ initial imbalance on the precision of the final MAIC estimates. The distributions of matching weights were inspected for extreme values, and are provided in Additional file 1: Figures S1, S2, and S3.

### Safety outcome—EPS

**Fig. 1:**
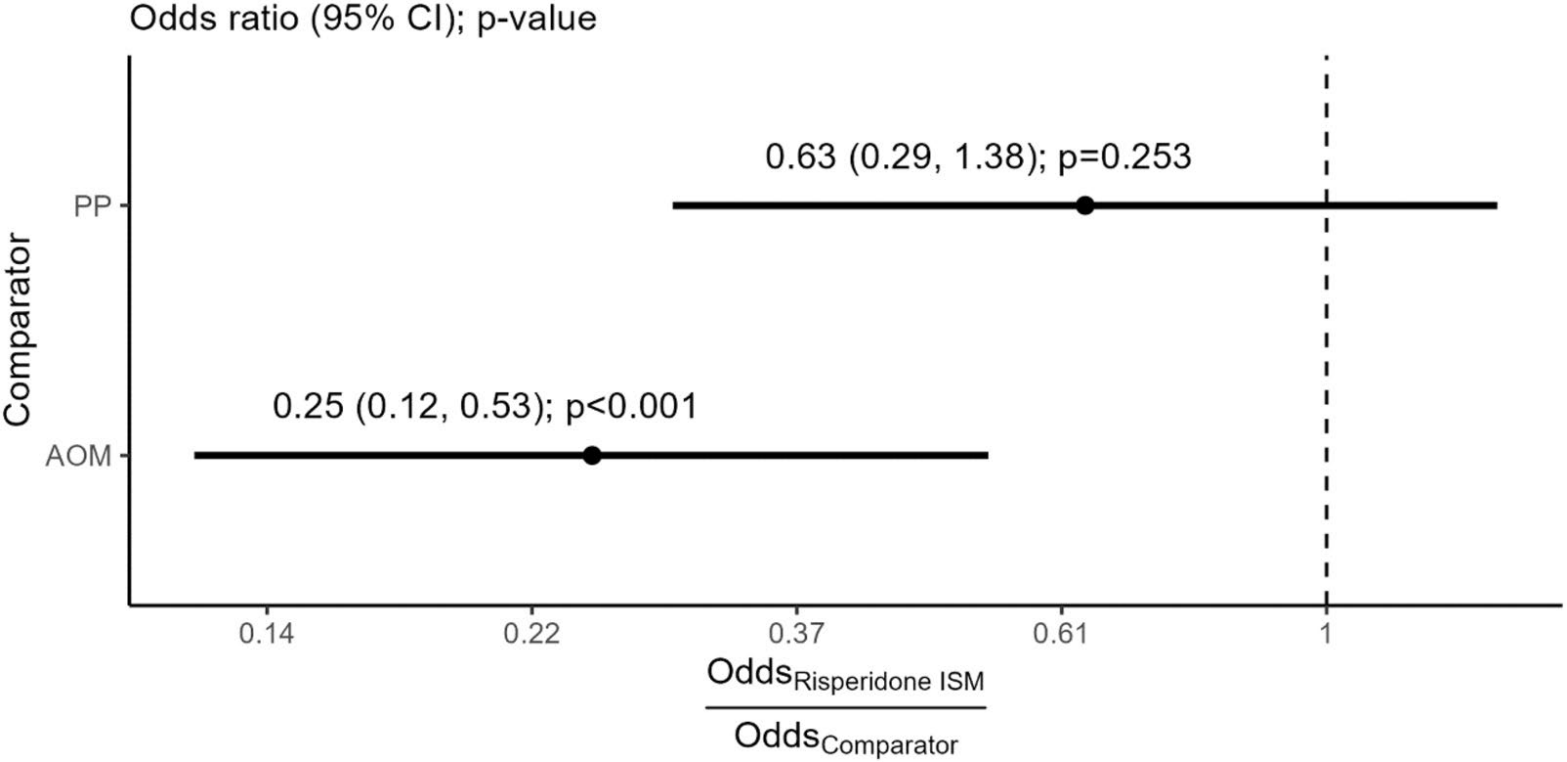
Extrapyramidal symptoms safety outcome—Base case estimates of Risperidone ISM versus comparator treatments. *AOM* aripiprazole monohydrate once-monthly, *CI* confidence interval, *PP* paliperidone palmitate

In the full population of the PRISMA-3 OLE, 4.2% of patients experienced EPS-related adverse events, compared to 6.4% of patients treated with PP (Gopal 2010) and 14.9% of patients treated with AOM (Kane 2012). Figure 1 presents unadjusted relative effects between Risperidone ISM and the comparator treatments, while detailed results are available in the Additional file 1: Table S4.

For the comparison versus PP, an OR (95% CI) of 0.63 (0.29, 1.38), *p* = 0.253 was estimated for Risperidone ISM, whereas an OR (95% CI) of 0.25 (0.12, 0.53), *p* < 0.001 was estimated versus AOM. As the comparator absolute effect is in the denominator, point estimates lower than 1 imply favorability for Risperidone ISM and lower odds for a patient to experience EPS-related adverse events. An additional sensitivity analysis for the comparison versus AOM (Kane 2012) using the pooled stabilized and stable cohorts of PRISMA-3 OLE yields similar results, with a statistically significant OR (95% CI) of 0.22 (0.09, 0.54), *p* = 0.001 in favor of Risperidone ISM (detailed results in Additional file 1: Table S5).

### Tolerability outcome—use of anticholinergic agents

**Fig. 2:**
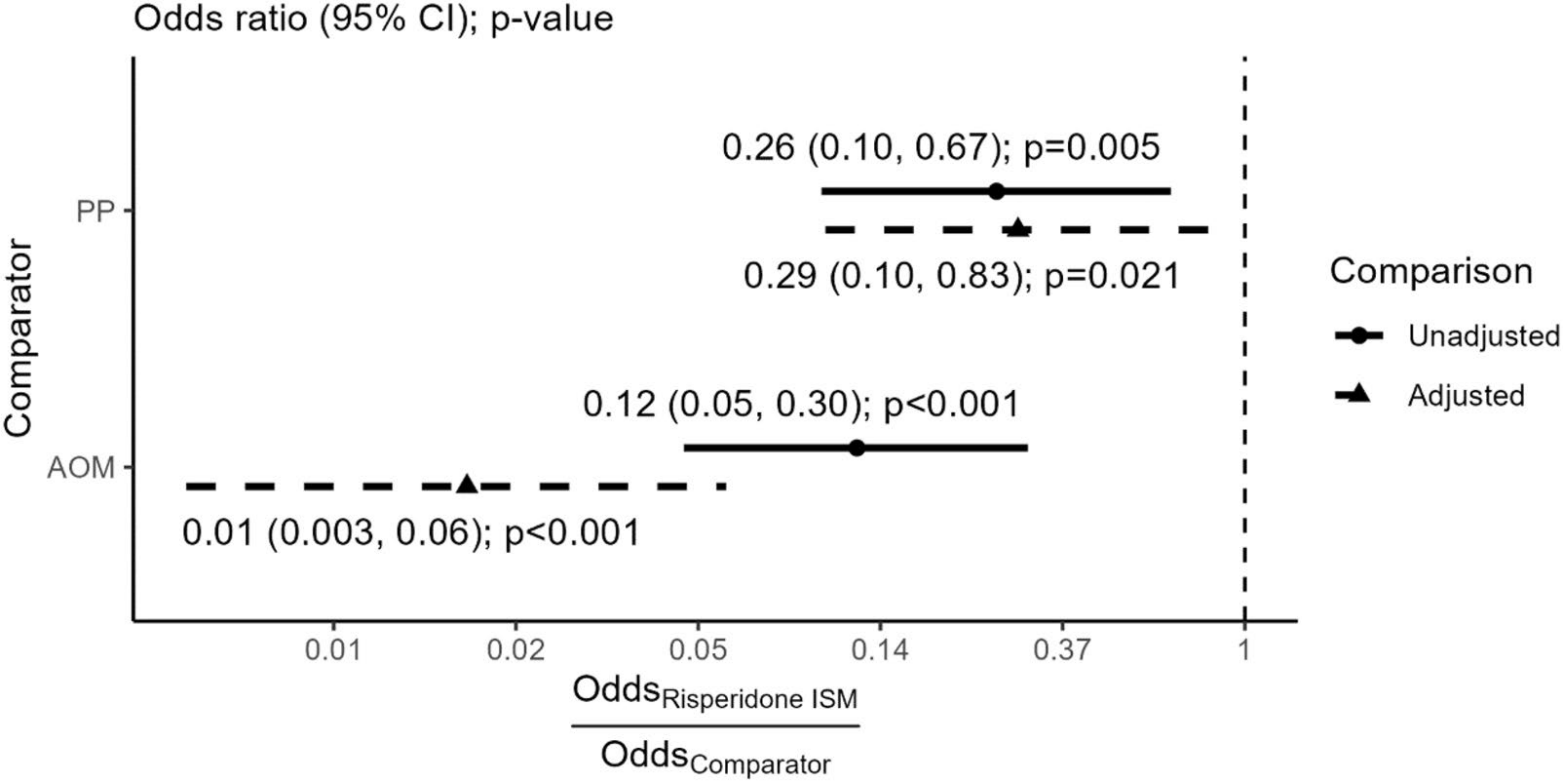
Anticholinergic agent use tolerability outcome—Base case estimates of Risperidone ISM versus comparator treatments. *AOM* aripiprazole monohydrate once-monthly, *CI* confidence interval, *PP* paliperidone palmitate

In the full population of the PRISMA-3 OLE, 2.3% of patients required administration of anticholinergic agents to alleviate EPS, compared to 8.5% in Gopal 2010, and 16.7% in the AOM arm of Kane 2012. Figure 2 presents both population-adjusted and unadjusted relative effects, with detailed results available in Additional file 1: Table S6.

In the MAIC of Risperidone ISM versus PP, population adjustment did not have a meaningful impact on the final estimate, shifting it from an unadjusted OR (95% CI) of 0.26 (0.10, 0.67), *p* = 0.005 to an adjusted OR (95% CI) of 0.29 (0.10, 0.83), *p* = 0.021. In contrast, adjustment in the comparison of Risperidone ISM to AOM substantially shifted the relative effect estimate towards a more favorable direction for the former, from an OR (95% CI) of 0.12 (0.05, 0.30), *p* < 0.001, to 0.01 (0.003, 0.06), *p* < 0.001. The sensitivity analysis which compared the pooled stabilized and stable cohorts of PRISMA-3 OLE versus Kane 2012 yielded similar results, with an adjusted OR of 0.01 (0.002, 0.05), *p* < 0.001, demonstrating superiority of Risperidone ISM over AOM (population matching results in Additional file 1: Table S7; detailed comparison results in Additional file: Table S8). In both analyses, population adjustments slightly widened confidence intervals compared to the unadjusted cases, which reflects the reduced precision following the reduction of the ESS.

## Discussion

The aim of this study was to indirectly compare the safety and tolerability profile of Risperidone ISM to LAI antipsychotics PP and AOM in schizophrenia patients treated in the maintenance setting, using MAIC methodology. The overall approach was consistent with UK (NICE DSU) and EU (EUnetHTA) best practices guidance for the conduct of MAICs [37, 38]. The outcomes analyzed were incidence of EPS and use of anticholinergic agents for the alleviation of EPS. Patient-level data from PRISMA-3 OLE were successfully matched in terms of baseline characteristics to the reported aggregate-level data of the Gopal 2010 and Kane 2012 studies, for the comparisons to PP and AOM respectively. The matched populations were subsequently used to produce adjusted relative effects, representing the effects we would expect to observe if the comparator studies had included a Risperidone ISM arm.

For the safety outcome of EPS, naïve, unanchored indirect comparisons demonstrated a statistically significant advantage of Risperidone ISM over AOM (OR [95% CI] 0.25 [0.12, 0.53], *p* < 0.001). In the comparison to PP, numerically favorable but not statistically significant results were observed for Risperidone ISM (OR [95% CI] 0.63 [0.29, 1.38], *p* = 0.253). In the analysis of the tolerability outcome of anticholinergic agent use, the MAIC demonstrated superiority of Risperidone ISM over both PP (OR [95% CI] 0.29 [0.10, 0.83], *p* = 0.021) and AOM (OR [95% CI] 0.01 [0.003, 0.06], *p* < 0.001), with a very high level of statistical significance in the latter case. Additional sensitivity comparisons to Kane 2012 that only used the stabilized and stable patients of PRISMA-3 OLE yielded similar conclusions with the base case analyses.

This indirect comparison has a number of limitations, both inherent to the MAIC methodology and specific to the particular setting. Due to the single-arm design of PRISMA-3 OLE, the MAIC was conducted under an unanchored framework, which assumes that all prognostic factors to be adjusted for are known and observed. This assumption is generally regarded as unlikely to hold, and its violation may induce bias in the results. MAICs further assume that the comparator populations that the IPD are matched to are representative of the patient populations for whom the treatment is intended in clinical practice. In contrast to PRISMA-3 OLE which had a fixed dosing regimen (patients were assigned to receive 75 mg or 100 mg of Risperidone ISM throughout the trial), the comparator studies had a flexible dosage protocol, which could bias tolerability results against Risperidone ISM. Substantial variability was observed across patient populations with regards to baseline PANSS and CGI-S scores, implying heterogeneity of disease severity (Table 1). For PANSS scores, this heterogeneity could follow from differences in trial protocols: In Gopal 2010, patients with PANSS ≤ 70 were enrolled into the DB phase prior to the OLE. In Kane 2012, patients with PANSS ≤ 80 were enrolled. In PRISMA-3, stable patients (de novo) with PANSS < 70 were recruited into the OLE, and patients with PANSS between 80 and 120 at screening were enrolled into the DB phase that preceded the OLE. In the comparison of PRISMA-3 OLE patients to those of Kane 2012, where variation in disease severity was more highly suspected, a subgroup sensitivity analysis was performed that validated the base case results. As PANSS and CGI-S were adjusted for in the MAIC, their imbalances contributed to the large reductions in the ESS. Some outcome definition inconsistency risks persist, as EPS are not clearly defined in the comparator studies, and Gopal 2010 mentions use of “anti-EPS medication” rather than anticholinergic agents. Lastly, an additional outcome of interest was the use of beta-blockers which are broadly used for the alleviation of anxiety-related schizophrenia symptoms and akathisia [49]. However, it was not reported in any comparator publication, and thus could not be pursued. In addition, some authors have pointed out that there are insufficient data to recommend beta-blocking drugs for akathisia [50, 51].

Given the persistent EPS risk associated with antipsychotics and the deleterious effects of EPS on patient quality of life [4, 6], it is crucial for novel treatments to demonstrate a superior safety profile with regards to this outcome. Anticholinergic agents, which constitute the primary treatment for the alleviation of antipsychotics-induced EPS, are known to cause a variety of distressing side-effects. As evidence is accumulating that their long-term use worsens the already compromised cognitive functions of schizophrenia patients and possibly even raises the risk of dementia [2, 12], clinicians gradually re-evaluate to prescribing them more prudently and only when absolutely needed to control EPS [8, 52, 53].

Based on the indirect comparative evidence, our findings demonstrate clinical benefit of Risperidone ISM when compared to other LAI antipsychotics, namely the monthly formulations of PP and AOM, in terms of lower EPS incidence and anticholinergic agent use. Although randomized controlled trials provide direct comparative evidence of the highest quality, indirect comparisons are valuable tools for clinicians and decision-makers in the absence of head-to-head comparisons, as past similar research in the field of antipsychotic treatment has shown [54, 55].

## Conclusions

In the indirect comparisons conducted, incidence of EPS was found to be statistically significantly lower in patients receiving Risperidone ISM than in those receiving AOM. Use of anticholinergic agents for the alleviation of EPS was also shown to be significantly lower in Risperidone ISM patients than in those receiving PP or AOM. Results from the sensitivity analyses comparing stabilized and stable patients receiving Risperidone ISM to those receiving AOM yielded similarly favorable conclusions in line with the base case analyses. Overall, this MAIC is in line with the favorable safety and tolerability results observed in the PRISMA-3 clinical trial investigating the long-term treatment of schizophrenia [24].

### Supplementary Information

**Additional file 1: Table S1**: Key characteristics of clinical trials identified for potential inclusion in the matching-adjusted indirect comparison. **Table S2**: Key trial inclusion and exclusion criteria. **Table S3**: Prognostic variable likelihood ratio test results for safety and tolerability outcomes in PRISMA-3 subgroups. **Table S4**: Detailed results of extrapyramidal symptoms safety base case outcome comparisons. **Table S5**: Detailed results of extrapyramidal symptoms safety outcome comparisons in the sensitivity analysis against Kane 2012. **Table S6**: Detailed results of anticholinergic agent use tolerability base case outcome comparisons. **Table S7**: Characteristics matching in Risperidone ISM versus Aripiprazole monohydrate once-monthly comparison, for the tolerability sensitivity analysis. **Table S8**: Detailed results of anticholinergic agent use in the tolerability outcome sensitivity analysis. **Figure S1**: Individual patient weights – Base case tolerability comparison to Gopal 2010. **Figure S2**: Individual patient weights – Base case tolerability comparison to Kane 2012. **Figure S3**: Individual patient weights – Sensitivity analysis tolerability comparison to Kane 2012.

## Data Availability

The datasets generated and/or analysed during the current study are not publicly available due to the sensitivity nature of individual patient data but may be made available by the corresponding author on reasonable request.

## Abbreviations

AOM: Aripiprazole monohydrate once-monthly
BMI: Body mass index
CGI-S: Clinical global impressions—severity scale
CI: Confidence interval
DB: Double-blind
DSU: Decision Support Unit
EPS: Extrapyramidal symptom(s)
ESS: Effective sample size
IPD: Individual patient data
LAI: Long-acting injectable
LRT: Likelihood ratio test
MAIC: Matching-adjusted indirect comparison
NICE: National Institute for Health and Care Excellence
NR: Not reported
OLE: Open-label extension
OR: Odds ratio
PANSS: Positive and negative syndrome scale
PCB: Placebo
PICOS: Population, intervention, comparator, outcome, study design
PP: Paliperidone palmitate
SD: Standard deviation
SLR: Systematic literature review
SMQ: Standardised MedDRA queries
TM: Transition/maintenance
