# Supplementary material for: Extrapyramidal adverse events and anticholinergics use after the long-term treatment of patients with schizophrenia with the new long-acting antipsychotic Risperidone ISM®: results from matching-adjusted indirect comparisons versus once-monthly formulations of Paliperidone palmitate and Aripiprazole monohydrate in 52-week studies
Source: Ann Gen Psychiatry. 2023 Sep 2;22:33. doi: 10.1186/s12991-023-00464-z (PMC10474682; doi:10.1186/s12991-023-00464-z)
Supplement: Supplementary file 1 — Additional file 1: Table S1: Key characteristics of clinical trials identified for potential inclusion in the matching-adjusted indirect comparison. Table S2: Key trial inclusion and exclusion criteria. Table S3: Prognostic variable likelihood ratio test results for safety and tolerability outcomes in PRISMA-3 subgroups. Table S4: Detailed results of extrapyramidal symptoms safety base case outcome comparisons. Table S5: Detailed results of extrapyramidal symptoms safety outcome comparisons in the sensitivity analysis against Kane 2012. Table S6: Detailed results of anticholinergic agent use tolerability base case outcome comparisons. Table S7: Characteristics matching in Risperidone ISM versus Aripiprazole monohydrate once-monthly comparison, for the tolerability sensitivity analysis. Table S8: Detailed results of anticholinergic agent use in the tolerability outcome sensitivity analysis. Figure S1: Individual patient weights – Base case tolerability comparison to Gopal 2010. Figure S2: Individual patient weights – Base case tolerability comparison to Kane 2012. Figure S3: Individual patient weights – Sensitivity analysis tolerability comparison to Kane 2012. [file 12991_2023_464_MOESM1_ESM.pdf]

Additional file

Literature review

The terms used for the search strategy were the following: (Schizophrenia and (acute or exacerbation or episode or relapse*) and (risperidone or paliperidone palmitate or aripiprazole or olanzapine pamoate) and (long-acting or long acting or injectable) and (efficacy or effectiveness or safety or outcome*)).m_titl.

Tables

Table S1: Key characteristics of clinical trials identified for potential inclusion in the matching-adjusted indirect comparison.

Study	Design	Treatment	Duration	Follow-up	Sample size
Hough 2010 (NCT00111189 DB phase) (1)	Phase III double-blind	PP versus placebo	24 weeks (mean)	Every 4 weeks	410
Gopal 2010 (NCT00111189 OLE phase) (2)	Phase III open-label	PP	1 year	Monthly	388
Kane 2012 (NCT00705783) (3)	Phase III double-blind	AOM versus placebo	1 year	Every 4 weeks	403
Fleischhacker 2012 (NCT00210717) (4)	Phase III double-blind	PP versus LAI-RIS	1 year	Monthly (PP) & biweekly (RIS-LAI)	749

Study	Design	Treatment	Duration	Follow-up	Sample size
Fleischhacker 2014 (NCT00706654) (5)	Phase III double-blind	AOM versus oral aripiprazole	38 weeks	Every 4 weeks	662
Naber 2015 (NCT01795547) (6)	Phase III open-label, rater-blinded	AOM versus PP	28 weeks	Monthly	295
PRISMA-3 OLE (NCT03870880) (7)	Phase III open-label	Risperidone ISM® 75mg versus 100mg	1 year	Monthly	215

Abbreviations: AOM, aripiprazole monohydrate once-monthly; DB, double-blind; LAI, long-acting injectable; OLE, open-label extension; PP, paliperidone palmitate; RIS, risperidone.

Table S2: Key trial inclusion and exclusion criteria.

Study	Inclusion criteria	Exclusion criteria
Hough 2010 & Gopal 2010 (NCT00111189) (1, 2)	• Patients who meet the diagnostic criteria for schizophrenia according to DSM-IV-TM for at least 1 year before screening<br>• PANSS score of <120<br>• BMI ≥ 15.0 kg/meter (m)2<br>• have resided at the same address for at least 30 days	• Patients unable to provide their own consent<br>• have been involuntarily committed to psychiatric hospitalization<br>• have primary, active DSM-IV-TM diagnosis other than schizophrenia<br>• who have a DSM-IV-TM diagnosis of active substance dependence within 3 months before screening<br>• have a history of treatment resistance as defined by failure to respond to 2 adequate trials (minimum of 4 weeks at a therapeutic dose) of different antipsychotic medications<br>• have a history of any severe preexisting gastrointestinal narrowing or inability to swallow the medication whole with water<br>• have a history of neuroleptic malignant syndrome<br>• are at significant risk of suicidal or violent behavior<br>• current presence of any significant or unstable medication condition<br>• treatment with any protocol disallowed therapies<br>• clinically significant result from screening laboratory or electrocardiogram
Kane 2012 (NCT00705783) (3)	• Subjects who are able to provide written informed consent and/or consent obtained from a legally acceptable representative (as required by the Institutional Review	• Subjects with a current DSM-IV-TR diagnosis other than schizophrenia, including schizoaffective disorder, major depressive disorder, bipolar disorder, delirium, dementia, or

Study	Inclusion criteria	Exclusion criteria
	Board/Institutional Ethics Committee [IRB/IEC]), prior to the initiation of any protocol-required procedures.<br>• Male and female subjects 18 to 60 years of age, inclusive, at time of informed consent.<br>• Subjects with a current diagnosis of schizophrenia as defined by Diagnostic and Statistical Manual of Mental Disorders, 4th edition text revision (DSM-IV-TR) criteria and a history of the illness for at least 3 years prior to screening.<br>• Subjects who, in the investigator's judgment, require chronic treatment with an antipsychotic medication.<br>• Subjects able to understand the nature of the study and follow protocol requirements, including the prescribed dosage regimens, tablet ingestion, IM depot injection, discontinuation of prohibited concomitant medications; who can read and understand the written word in order to complete patient-reported outcomes measures; and who can be reliably rated on assessment scales	amnestic or other cognitive disorders. Also, subjects with borderline, paranoid, histrionic, schizotypal, schizoid, or antisocial personality disorder.<br>• Subjects with schizophrenia that are considered resistant/refractory to antipsychotic treatment by history or response only to clozapine.<br>• Subjects with a significant risk of violent behavior or a significant risk of committing suicide based on history or investigator's judgment.<br>• Subjects who currently meet DSM-IV-TR criteria for substance dependence, including alcohol and benzodiazepines, but excluding caffeine and nicotine; or 2 positive drug screens for cocaine.<br>• Subjects who are known to be allergic, intolerant, or unresponsive to prior treatment with aripiprazole or other quinolinones; or hypersensitivity to antipsychotic agents.<br>• Subjects with uncontrolled thyroid function abnormalities.<br>• Subjects with a history of seizures, neuroleptic malignant syndrome, clinically significant tardive dyskinesia, or other medical condition that would expose them to undue risk or interfere with study assessments.<br>• Subjects who are involuntary incarcerated.<br>• Subjects who have used an investigational agent within 30 days of screening or prior participation in a clinical study with aripiprazole IM depot.

Study	Inclusion criteria	Exclusion criteria
		• Subjects with clinically significant abnormalities in laboratory test results, vital signs, or electrocardiogram results; and subjects hospitalized for more than 30 days in the 90 days prior to Phase 1<br>• Subjects who fail to wash-out from prohibited concomitant medications, including the use of CYP2D6 or CYP3A4 inhibitors or CYP3A4 inducers, antipsychotics, antidepressants (including monoamine oxidase inhibitors [MAOI}), and mood stabilizers during screening and/or Phase 1
Fleischhacker 2012 (NCT00210717) (4)	• A diagnosis of schizophrenia (disorganized, catatonic, paranoid, residual, or undifferentiated types) according to the Diagnostic and Statistical Manual of Mental Disorders, 4th edition (DSM IV) for at least 1 year before the screening evaluation<br>• a total PANSS score of 60 to 120 at screening and baseline (pre-treatment) evaluations<br>• a body mass index (BMI [weight (kilograms)]/[height (meters)]^2) of at least 15.0 kg/m^2	• A primary active DSM-IV Axis I diagnosis other than schizophrenia<br>• a decrease of 25% or more in the total PANSS score between screening and baseline evaluations<br>• a DSM-IV diagnosis of active substance dependence within 3 months of screening evaluation<br>• a history of treatment resistance as defined by failure to respond to 2 adequate trials of different antipsychotic medications<br>• a woman who is pregnant, breast-feeding, or planning to become pregnant during the study period
Fleischhacker 2014	• Subjects who are able to provide written informed consent and/or consent obtained from a legally acceptable representative (as required by Institutional Review	• Subjects with a current DSM-IV-TR diagnosis other than schizophrenia, including schizoaffective disorder, major depressive disorder, bipolar disorder, delirium, dementia, amnestic, or other cognitive disorders. Also, subjects with borderline, paranoid, histrionic, schizotypal, schizoid, or antisocial personality disorder.

Study	Inclusion criteria	Exclusion criteria
(NCT00706654)<br><br>(5)	Board/Independent Ethics Committee [IRB/IEC]), prior to the initiation of any protocol-required procedures.<br>• Male and female subjects 18 to 60 years of age, inclusive, at time of informed consent.<br>• Subjects with a current diagnosis of schizophrenia as defined by Diagnostic and Statistical Manual of Mental Disorders, version 4, Text Revision (DSM-IV-TR) criteria and a history of the illness for at least 3 years prior to screening.<br>• Subjects who, in the investigator's judgment, require chronic treatment with an anti-psychotic medication.<br>• Subjects able to understand the nature of the study and follow protocol requirements, including the prescribed dosage regimens, tablet ingestion, IM depot injection, discontinuation of prohibited concomitant medications, who can read and understand the written word in order to complete patient-reported outcome measures, and who can be reliably rated on assessment scales	• Subjects with schizophrenia that are considered resistant/refractory to antipsychotic treatment by history or response only to clozapine.<br>• Subjects with a significant risk of violent behavior or a significant risk of committing suicide based on history or investigator's judgment.<br>• Subjects who currently meet DSM-IV-TR criteria for substance dependence; including alcohol and benzodiazepines, but excluding caffeine and nicotine, or 2 positive drug screens for cocaine.<br>• Subjects who are known to be allergic, intolerant, or unresponsive to prior treatment with aripiprazole or other quinolinones, or hypersensitivity to anti-psychotic agents, including aripiprazole.<br>• Subjects with a history of neuroleptic malignant syndrome or clinically significant tardive dyskinesia at screening.<br>• Subjects with uncontrolled thyroid function abnormalities.<br>• Subjects with a history of seizures, neuroleptic malignant syndrome, clinically significant tardive dyskinesia, or other medical condition that would expose the subject to undue risk or interfere with study assessments.<br>• Subjects who are involuntarily incarcerated.<br>• Subjects who have undergone electroconvulsive therapy within 180 days of entry into Phase 2.

Study	Inclusion criteria	Exclusion criteria
		• Subjects who have used an investigational agent within 30 days of screening; and prior participation in a clinical study with aripiprazole IM depot.<br>• Subjects with clinically significant abnormalities in laboratory test results, vital signs, or ECG results.<br>• Subjects hospitalized for more than 30 days in the 90 days prior to Phase 1 (or Phase 2 for subjects bypassing Phase 1)<br>• Subjects requiring more than 1 benzodiazepine beyond screening (eg, lorazepam and oxazepam).<br>• Subjects who fail to wash-out from prohibited concomitant medications, including the use of CYP2D6 or CYP3A4 inhibitors or CYP3A4 inducers, antipsychotics, antidepressants (including monoamine oxidase inhibitors [MAOI]), and mood stabilizers, during screening and Phase 1
Naber 2015 (NCT01795547) (6)	• The patient has schizophrenia, diagnosed according to DSM-IV-TR®.<br>• The patient has a CGI-S score from mildly ill to markedly ill at the Screening and Baseline Visit(s).<br>• The patient is in need of a change in the current antipsychotic treatment and in the judgement of the investigator the patient would benefit from an extended treatment with a once-monthly formulation.	• The patient has any current psychiatric disorder or Axis I disorder (DSM-IV® criteria) other than schizophrenia established as the primary diagnosis.<br>• The patient is experiencing acute exacerbation of psychotic symptoms at the Screening Visit or between the Screening and Baseline Visits.<br>• The patient in the investigator's judgment has shown significant intolerance and/or lack of efficacy to oral aripiprazole, paliperidone or risperidone.<br>• The patient is at significant risk of harming himself/herself or others according to the investigator's judgement or according to Columbia-Suicide Severity Rating Scale (C-SSRS).

Study	Inclusion criteria	Exclusion criteria
	• The patient agrees to protocol-defined use of effective contraception	• The patient has a history of neuroleptic malignant syndrome.<br>• The patient has or has had significant medical condition that would expose him or her to an undue risk of a significant adverse event or interfere with assessments of safety or efficacy during the course of the study including, but not limited to neurological, hepatic, renal, metabolic, haematological, immunological, gastrointestinal, pulmonary, or cardiovascular disorders.<br>• The patient has a disease or takes medication that could, in the investigator's opinion, interfere with the assessments of safety, tolerability, or efficacy, or interfere with the conduct or interpretation of the study.<br>• The patient is, in the investigator's opinion, unlikely to comply with the protocol or is unsuitable for any reason

Study	Inclusion criteria	Exclusion criteria
PRISMA-3 OLE<br>(NCT03870880)<br>(7)	Rollover patients:<br>• Has completed scheduled participation in the double blind segment of the study PRISMA-3, through to the end of the treatment period and including the end-of-treatment visit<br>• Continues to require long-term treatment with an antipsychotic medication, in the opinion of the investigator<br>• Continues to meet contraceptive requirements of the study PRISMA-3<br>• Is willing to participate in the extension segment of the study and remains capable of providing informed consent<br>• Continues to reside in a stable living situation, in the opinion of the investigator<br>• Continues to have an identified reliable informant, in the opinion of the investigator<br><br>De novo patients:<br>• Capable of providing informed consent<br>• Age ≥ 18 and ≤ 65 years old<br>• On a stable dose of oral risperidone from 4 to 6 mg daily as maintenance therapy for at least the last 4 weeks	Rollover patients:<br>• Missed more than 1 scheduled study visit during participation in the double blind segment of study PRISMA-3<br>• Had an abnormal clinical laboratory value, vital sign, or electrocardiogram finding during participation in the main part of the study that, in the opinion of the investigator, was clinically relevant, related to study drug, and would compromise the well-being of the patient in the extension segment<br>• Had a clinically significant or unstable medical illness/condition/disorder during the main part of the study that would be anticipated, in the investigator's opinion, to potentially compromise patient safety in the extension segment<br>• Is taking or is anticipated to require any prohibited concomitant medication<br>• Pregnant, lactating, or breastfeeding<br>• Any contraindication for continued IM injections (e.g., treatment with anticoagulant)<br>• Inadequate gluteal or deltoid musculature or excessive fat, as determined by the investigator, that would interfere with IM study drug injections<br>• Study site personnel and/or persons employed by the investigator or study site or is an immediate family member of such persons<br><br>De novo patients:

Study	Inclusion criteria	Exclusion criteria
	prior/before screening/baseline and would potentially benefit from conversion to an extended release injectable, in the opinion of the investigator<br>• Current diagnosis of schizophrenia, according to the Diagnostic and Statistical Manual of Mental Disorders, Fifth Edition (DSM-5) criteria that is clinically stable as evidenced by:<br>○ No hospitalizations for acute exacerbations of schizophrenia and psychiatrically stable without significant symptom exacerbation over the last 3 months before screening based on the investigator's judgment<br>○ PANSS total score < 70 at screening<br>○ CGI-S score of ≤ 3 (mild) at screening<br>• Has previously had a clinically significant beneficial response (improvement in schizophrenia symptoms), as determined by the investigator, to treatment with an antipsychotic medication other than clozapine<br>• At least 2 years elapsed since initial onset of active-phase schizophrenia symptoms	• History of proven inadequate clinical response to treatment with therapeutic doses (with good compliance) of risperidone or paliperidone<br>• History of treatment resistance, defined as failure to respond to 2 discrete adequate trials (≥ 4 weeks with an adequate dose) of 2 different antipsychotic medications; history of clozapine use (exception: use was not because of treatment resistance or refractory psychotic symptoms)<br>• Known or suspected intolerance of or allergy or hypersensitivity to risperidone, paliperidone, or any of the excipients in the IM formulations of these<br>• History of neuroleptic malignant syndrome, clinically significant tardive dyskinesia or tardive dystonia<br>• History of any other medical condition that is considered to pose any unjustifiable risk or interfere with study assessments<br>• Clinically significant extrapyramidal symptoms at screening or baseline<br>• At significant risk of suicidal, homicidal or violent ideation or behavior, by history or as clinically assessed by the investigator at screening visit<br>• Answer of "yes" on item 4 or on item 5 of the Columbia-Suicide Severity Rating Scale (C-SSRS) (ideation) with the most recent episode occurring within the past 2 months, or answer "yes" to any of the 5 items (behavior) with an episode occurring within the last year

Study	Inclusion criteria	Exclusion criteria
	• Subject is outpatient; not hospitalized for worsening of schizophrenia within the last 3 months (hospitalization for social management within this time period is acceptable)<br>• Medically stable over the last month prior to screening based on the investigator's judgment<br>• BMI of 18.5 to 40.0 kg/m2 (inclusive) at screening<br>• Agrees to discontinue prohibited medications as applicable and as clinically indicated according to investigator instructions<br>• Dosages of all permitted medications are considered to have been stable (with the exception of medication to be used on an as-needed basis) for ≥ 2 weeks prior to the baseline visit and to remain stable during participation in this study<br>• Resides in a stable living situation, in the opinion of the investigator<br>• Has an identified reliable informant, in the opinion of the investigator<br>• Meets the contraceptive criteria stablished in the study	• Current diagnosis or a history of substance use disorder according to DSM-5 criteria within 6 months prior to the screening visit (with the exception of tobacco, mild cannabis, or mild alcohol use disorder) or a positive drug screen test (with the exception of cannabis) verified by repeat testing<br>• Lifetime history of diagnosis of schizoaffective disorder or bipolar disorder<br>• Clinically significant comorbid neuropsychiatric disorders<br>• Clinically significant or unstable medical illness/condition/disorder that would be anticipated, in the investigator's opinion, to potentially compromise patient safety or adversely affect the evaluation of efficacy<br>• Laboratory abnormality that, in the opinion of the investigator, would compromise the well-being of the patient, or any of the following laboratory abnormalities at screening or baseline<br>• Pregnant, lactating, or breastfeeding<br>• Inadequate gluteal or deltoid musculature or excessive fat, as determined by the investigator, that would interfere with IM study drug injections<br>• Any contraindication for IM injections<br>• Receipt of any long-acting antipsychotic medication by IM injection within 60 days before screening<br>• Current involuntary hospitalization or incarceration<br>• Hospitalized for more than 30 days during the 90 days before screening

Study	Inclusion criteria	Exclusion criteria
	• Agrees not to post any personal medical data related to the study or information related to the study on any website or social media site during the study duration	• Participation in another clinical study in which the patient received an experimental or investigational drug or agent within 6 months before screening<br>• Participation in a clinical study with Risperidone ISM® within 12 months before screening<br>• Study site personnel and/or persons employed by the investigator or study site or is an immediate family member of such persons<br>• Patients taking or anticipated to require any prohibited concomitant medication

Table S3: Prognostic variable likelihood ratio test results for safety and tolerability outcomes in PRISMA-3 subgroups.

Baseline characteristic	PRISMA-3 cohort	EPS	Anticholinergic agent use
Age	All	0.834	0.544
	Stabilized[a] & stable[b]	0.557	**0.055**
Sex	All	0.737	0.966
	Stabilized & stable	0.829	0.879
Race	All	0.903	**< 0.001**
	Stabilized & stable	0.934	**< 0.001**
BMI	All	0.441	0.976
	Stabilized & stable	0.520	0.670
Age at diagnosis	All	0.337	0.422
	Stabilized & stable	0.284	0.775
PANSS	All	0.152	**0.002**
	Stabilized & stable	0.547	**0.009**
CGI-S	All	0.158	**0.007**
	Stabilized & stable	0.221	**0.040**

Abbreviations: BMI, body mass index; CGI-S, clinical global impressions – severity scale; DB double-blind; EPS, extrapyramidal symptom; OLE, open-label extension; PANSS, positive and negative syndrome scale. [a] Stabilized patients, patients treated with Risperidone ISM in the DB phase who continued to receive monthly Risperidone ISM in the OLE phase at the same dose (75 or 100 mg) as during the DB phase; [b] Stable patients, newly enrolled patients (de novo) who were on a previous stable

maintenance dose of oral risperidone.

Notes: Numbers in bold characters denote *p*-values lower than 0.1, which is the statistical significance threshold assumed in the prognostic factor selection process.

Table S4: Detailed results of extrapyramidal symptoms safety base case outcome comparisons.

**Analysis**	**Summary statistic**	**Effect**	**Estimate**	**95% CI**	***p*-value**
PRISMA-3 OLE versus Gopal 2010	Odds	Risperidone ISM	0.044	(0.022, 0.085)	< 0.001
	Odds	PP	0.069	(0.046, 0.103)	< 0.001
	Odds ratio	Risperidone ISM versus PP	0.634	(0.291, 1.380)	0.253
PRISMA-3 OLE versus Kane 2012	Odds	Risperidone ISM	0.044	(0.022, 0.085)	< 0.001
	Odds	AOM	0.175	(0.125, 0.244)	< 0.001
	Odds ratio	Risperidone ISM versus AOM	0.250	(0.118, 0.528)	< 0.001

Abbreviations: AOM, aripiprazole monohydrate once-monthly; CI, confidence interval; OLE, open-label extension; PP, paliperidone palmitate.

Table S5: Detailed results of extrapyramidal symptoms safety outcome comparisons in the sensitivity analysis against Kane 2012.

Summary statistic	Effect	Estimate	95% CI	*p*-value
Odds	Risperidone ISM	0.039	(0.017, 0.088)	< 0.001
Odds	AOM	0.175	(0.125, 0.244)	< 0.001
Odds ratio	Risperidone ISM versus AOM	0.223	(0.092, 0.539)	0.001

Abbreviations: AOM, aripiprazole monohydrate once-monthly; CI, confidence interval; OLE, open-label extension.

Table S6: Detailed results of anticholinergic agent use tolerability base case outcome comparisons.

MAIC analysis	Summary statistic	Effect	Comparison	Estimate	95% CI	*p*-value
PRISMA-3 OLE versus Gopal 2010	Odds	Risperidone ISM	Adjusted	0.027	(0.010, 0.072)	< 0.001
	Odds	Risperidone ISM	Unadjusted	0.024	(0.010, 0.058)	< 0.001
	Odds	PP	Unadjusted	0.093	(0.065, 0.133)	< 0.001
	Odds ratio	Risperidone ISM versus PP	Adjusted	0.288	(0.100, 0.827)	0.021

	Odds ratio	Risperidone ISM versus PP	Unadjusted	0.256	(0.098, 0.666)	0.005
PRISMA-3 OLE versus Kane 2012	Odds	Risperidone ISM	Adjusted	0.003	(0.001, 0.011)	< 0.001
	Odds	Risperidone ISM	Unadjusted	0.024	(0.010, 0.058)	< 0.001
	Odds	AOM	Unadjusted	0.201	(0.146, 0.277)	< 0.001
	Odds ratio	Risperidone ISM versus AOM	Adjusted	0.014	(0.003, 0.058)	< 0.001
	Odds ratio	Risperidone ISM versus AOM	Unadjusted	0.119	(0.046, 0.304)	< 0.001

Abbreviations: AOM, aripiprazole monohydrate once-monthly; CI, confidence interval;; OLE, open-label extension; PP, paliperidone palmitate.

Table S7: Characteristics matching in Risperidone ISM versus Aripiprazole monohydrate once-monthly comparison, for the tolerability sensitivity analysis.

**Study**	**PRISMA-3 OLE (pooled stable & stabilized cohorts)**		**Kane 2012 (AOM arm)**
Characteristic	Pre-matching (*N* = 160)	Post-matching (ESS = 26)	(*N* = 269)
Age [Mean (SD)]	39.4 (11.2)	40.1 (11.0)	40.1 (11.0)
Baseline PANSS total score [Mean]	67.5	54.5	54.5
Baseline CGI-S total score [Mean]	3.2	2.9	2.9
Race: white [%]	86.2	56.5	56.5

Abbreviations: AOM, aripiprazole monohydrate once-monthly; CGI-S, clinical global impressions - severity scale; ESS, effective sample size; OLE, open-label extension; PANSS, positive and negative syndrome scale; SD, standard deviation.

Table S8: Detailed results of anticholinergic agent use in the tolerability outcome sensitivity analysis.

Summary statistic	Effect	Comparison	Estimate	95% CI	*p*-value
Odds	Risperidone ISM	Adjusted	0.002	(0.000, 0.009)	< 0.001
Odds	Risperidone ISM	Unadjusted	0.019	(0.006, 0.060)	< 0.001
Odds	AOM	Unadjusted	0.201	(0.146, 0.277)	< 0.001
Odds ratio	Risperidone ISM versus AOM	Adjusted	0.010	(0.002, 0.047)	< 0.001
Odds ratio	Risperidone ISM versus AOM	Unadjusted	0.095	(0.029, 0.312)	< 0.001

Abbreviations: AOM, aripiprazole monohydrate once-monthly; CI, confidence interval; OLE, open-label extension; PP, paliperidone palmitate.

Figures

The distribution of the population matching weights is presented in Supplementary Figures 1, 2, and 3. According to MAIC methodology, visual inspection helps identify extreme cases of patients with large weights, which is caused if their baseline characteristics are significantly different from those of the population they were matched into. Most patients have weights lower than 5 in all comparisons. A few patients have large values (e.g.: above 10), which cause reductions in the effective sample size (ESS).

Figure S1: Individual patient weights – Base case tolerability comparison to Gopal 2010.

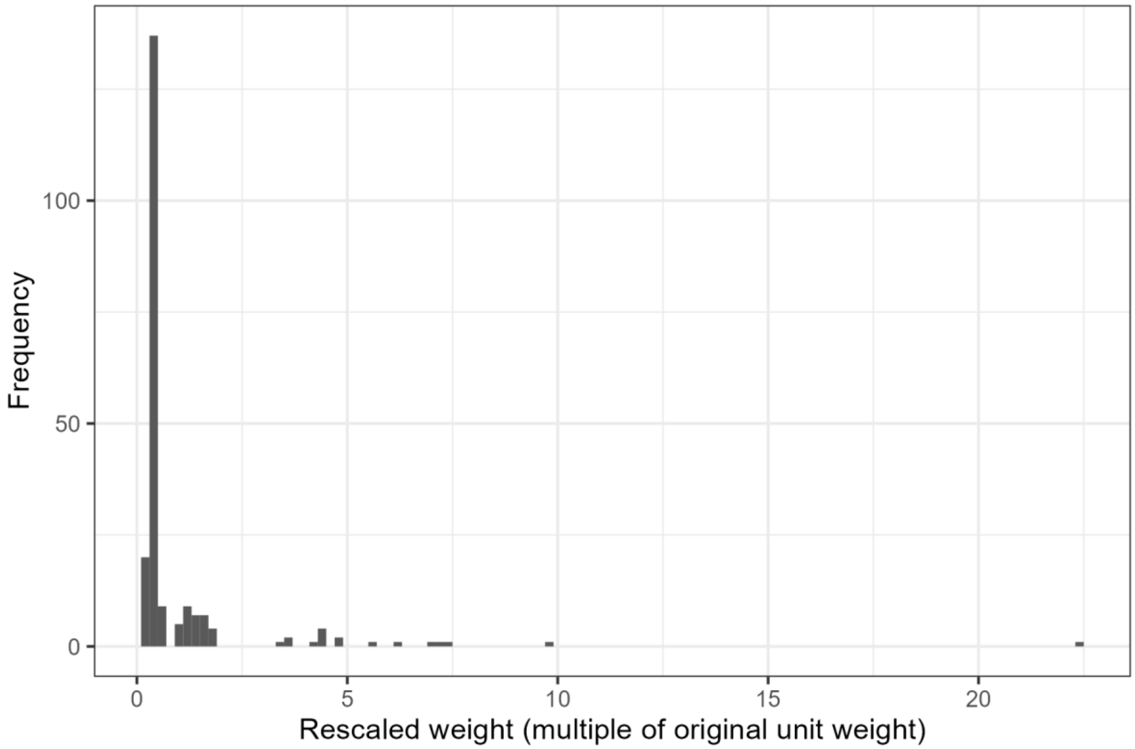

Figure S2: Individual patient weights – Base case tolerability comparison to Kane 2012.

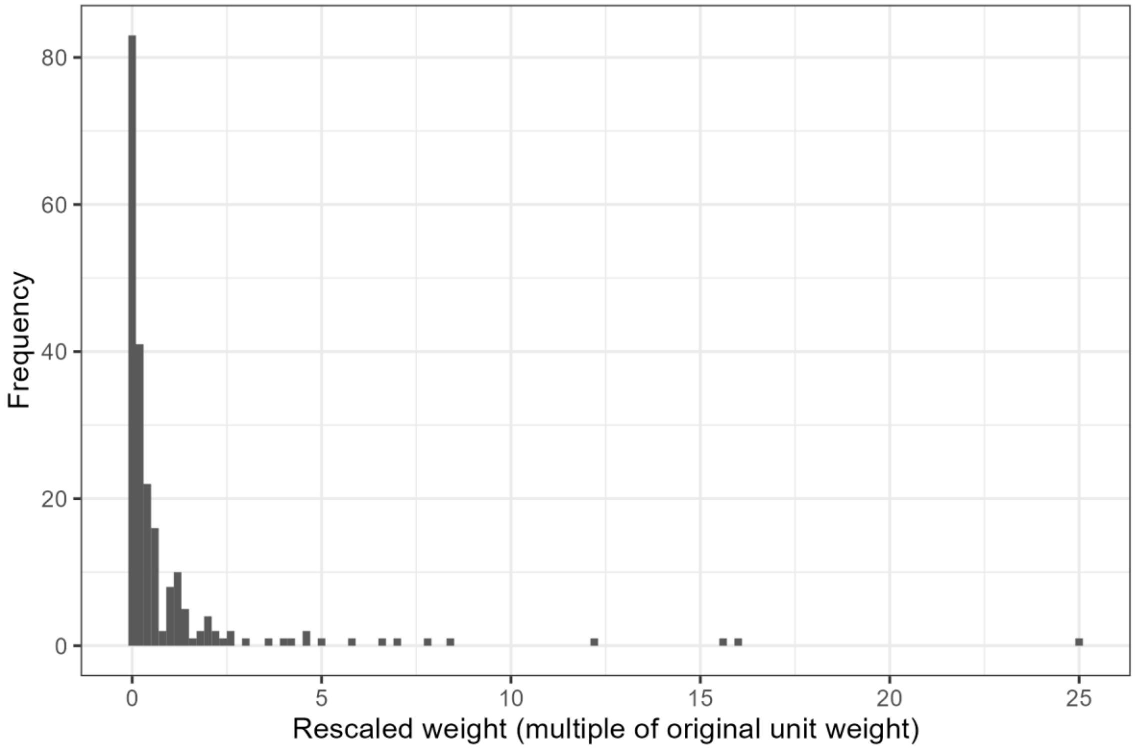

Figure S3: Individual patient weights – Sensitivity analysis tolerability comparison to Kane 2012.

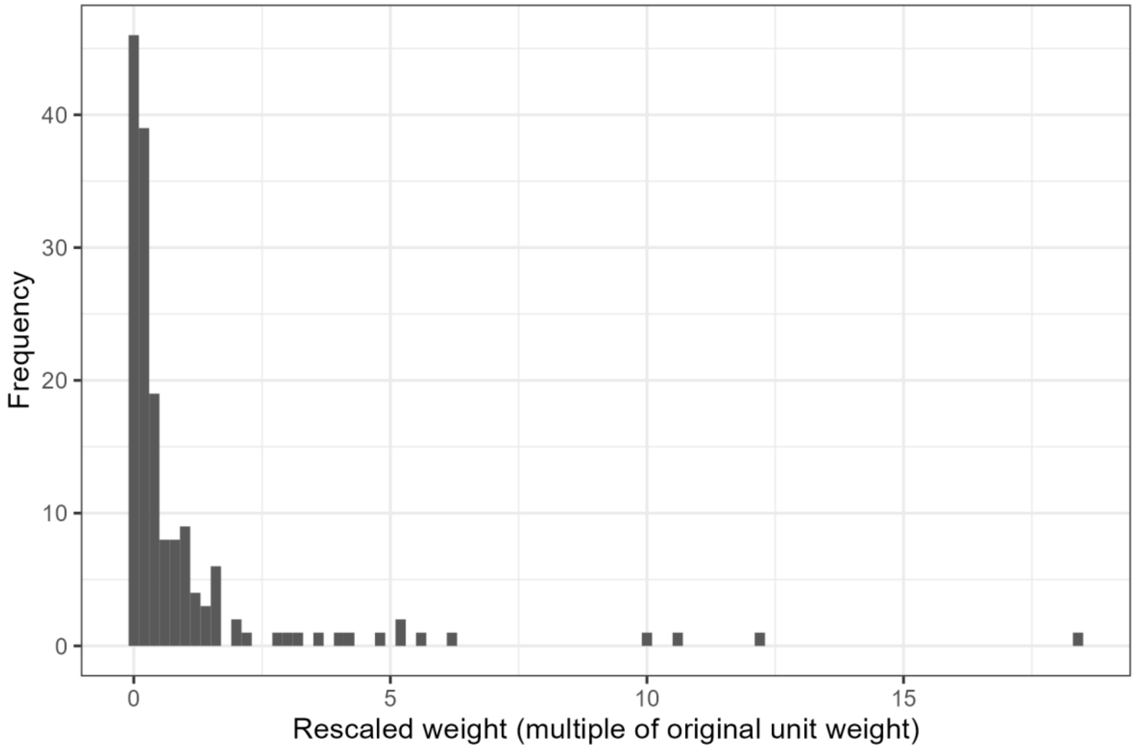

References

1. Hough D, Gopal S, Vijapurkar U, Lim P, Morozova M, Eerdekens M. Paliperidone palmitate maintenance treatment in delaying the time-to-relapse in patients with schizophrenia: a randomized, double-blind, placebo-controlled study. Schizophrenia research. 2010;116(2-3):107-17.

2. Gopal S, Vijapurkar U, Lim P, Morozova M, Eerdekens M, Hough D. A 52-week open-label study of the safety and tolerability of paliperidone palmitate in patients with schizophrenia. Journal of Psychopharmacology. 2011;25(5):685-97.

3. Kane JM, Sanchez R, Perry PP, Jin N, Johnson BR, Forbes RA, et al. Aripiprazole intramuscular depot as maintenance treatment in patients with schizophrenia: a 52-week, multicenter, randomized, double-blind, placebo-controlled study. The Journal of clinical psychiatry. 2012;73(5):1339.

4. Fleischhacker WW, Gopal S, Lane R, Gassmann-Mayer C, Lim P, Hough D, et al. A randomized trial of paliperidone palmitate and risperidone long-acting injectable in schizophrenia. The The International Journal of Neuropsychopharmacology. 2012;15(1):107-18.

5. Fleischhacker WW, Sanchez R, Perry PP, Jin N, Peters-Strickland T, Johnson BR, et al. Aripiprazole once-monthly for treatment of schizophrenia: double-blind, randomised, non-inferiority study. The British Journal of Psychiatry. 2014;205(2):135-44.

6. Naber D, Hansen K, Forray C, Baker RA, Sapin C, Beillat M, et al. Qualify: a randomized head-to-head study of aripiprazole once-monthly and paliperidone palmitate in the treatment of schizophrenia. Schizophrenia research. 2015;168(1-2):498-504.

7. Filts Y, Litman RE, Martínez J, Anta L, Naber D, Correll CU. Long-term efficacy and safety of once-monthly Risperidone ISM® in the treatment of schizophrenia: Results from a 12-month open-label extension study. Schizophrenia Research. 2022;239:83-91.
